# Four-Port Dual-Band Multiple-Input Multiple-Output Dielectric Resonator Antenna for Sub-6 GHz 5G Communication Applications

**DOI:** 10.3390/mi13112022

**Published:** 2022-11-19

**Authors:** Upesh Patel, Trushit Upadhyaya

**Affiliations:** Electronics and Communication Department, Chandubhai S. Patel Institute of Technology, Charotar University of Science and Technology (CHARUSAT), Changa 388421, India

**Keywords:** dielectric resonator, MIMO communication, 5G antenna, Sub-6 GHz communication, WiMAX communication

## Abstract

A four-port dielectric resonator (DR)-based multiple-input multiple-output (MIMO) antenna is presented for sub-6 GHz MIMO communication. The dielectric resonator was fed through aperture feeding to achieve dual-band resonance. The DRA has the operating modes of $TE_{01\delta }$ and $TE_{10\delta }\;$at 3.3 GHz and 3.9 GHz, respectively. The engineered antenna has port isolation of higher than 20 dB at the target frequencies without the employment of an extra isolation mechanism. Full-wave high-frequency simulation software was employed for the simulation computation. The antenna has a peak gain of 5.8 dBi and 6.2 dBi, and an efficiency of 88.6% and 90.2% at 3.3 GHz and 3.9 GHz, respectively. The proposed resonator has good MIMO diversity parameters. The optimal envelope correlation coefficient (ECC) is 0.01, channel capacity loss (CCL) is 0.1 bits/sec/Hz, and the total active reflection coefficient (TARC) is −22.46. The DRA elements are aligned orthogonally with adequate displacement for achieving polarization diversity and spatial diversity. The antenna delivers its applications in Sub-6 GHz 5G and WiMAX communications.

## 1. Introduction

The significantly rising requirement for real-time internet traffic has triggered the requirement for fast and efficient communication. The present data streaming need has to overcome the restrictions offered by 3G and 4G communications. While there is a strict constraint on channel allocation numbers, there also exist severe limitations in multi-user accommodation due to co-channel interference. The substantial evolution in wireless communication has triggered 5G communication having the capability to support data rates up to multiple gigabits per second. Each advanced generation has presented a multifold growth in communication technology and a higher data rate [1,2]; low latency and delay are offered with 5G communication in comparison with 4G communication. The multiple-input multiple-output (MIMO) technology provides increased channel capacity, mitigates multipath fading, and provides faster communication. MIMO provides seamless communication through optimum spectrum efficiency, and through effective channel allocation, it also provides more mobile users.

The 5G communication technology inducts an array of antennas. A MIMO communication tames radio propagation issues by communicating the data over manifold transmit and multiple receive antennas. The multiple fading inconsistency affects the data transmitted over each antenna and hence the fading received in each channel differs in nature. At present, there is a wide spectrum of multiplexing techniques available for giving a high degree of freedom in MIMO communication. The 5G mobile communication has a strong constraint of physical space for incorporating the antennas. Planar resonators are a feasible choice for the sustenance of multiple antenna systems. Patch resonators provide appropriateness in manufacturing and cost-competitiveness. The patch antennas suffer significantly in terms of providing good antenna parameters, especially bandwidth and gain. The standard patch antenna employs high dielectric loss material to provide cost-competitiveness and hence there has to be a balance in the trade-off between antenna parameters and cost-effectiveness. The dielectric resonator antennas (DRAs) are capable of providing enhanced radiation efficiency when they are fed appropriately. The DRA design with proper electrical and mechanical attributes leads to greater radiation efficiency. The literature presents a few of the DRAs designed for 5G MIMO communication [3,4,5,6,7,8,9,10].

MIMO antennas are typically expected to give low mutual coupling between ports. The high isolation or low correlation ensures better radiation and diversity performance in MIMO systems. These antenna properties are expected in a space-constrained environment and it is extremely important to have electrical compactness in the antenna design. This makes the MIMO antenna design exciting. There is significant literature available relating to the improvement of MIMO antenna port isolation through the decoupling mechanism [11,12,13,14,15,16,17,18,19,20]. In the absence of the decoupling mechanism, port isolation can be achieved by the appropriate placement of the resonators which are fed through transmission lines. Internal mode decoupling is frequently used for achieving better port isolation. The engineered resonators will guarantee opposite current flow directions in the resonator to avoid a strong correlation between the resonating elements. The WiMAX communication standard provides broadband services to end-users. It is capable of providing a higher data rate compared to WiFi communication over a longer distance. MIMO communication requires multiple resonant frequencies and there has been substantial research in MIMO technology [21,22,23,24,25,26,27]. The recent literature presents state-of-the-art MIMO antennas [28,29,30,31,32]. The effective use of a 3–300 GHz underutilized spectrum may aid in providing a reasonable trade-off between user coverage and channel capacity, as presented by 3G and 4G communication. By engineering the dielectric resonator, improved antenna parameters viz. directivity, gain, bandwidth, and cross-pol isolation can be achieved. The adequate selection of antenna dimensions and appropriate feeding mechanisms can ensure the excitation of multiple resonant modes. The novelty of the presented design is that by maneuvering the dielectric resonator two modes viz. $TE_{01\delta }$ and $TE_{10\delta }\;$are excited in antenna dual-band operation. The antenna also provides electrical compactness for integration in the RF circuit design. The presented antenna has an excellent application in Sub-6GHz and WiMAX Communication. Section 2 describes antenna design, the antenna sensitivity analysis is presented in Section 3 to show the effects of physical parameters on antenna resonance, and Section 4 and Section 5 of the manuscript incorporate implementation on 4 × 4 MIMO design followed by a critical discussion of the results.

## 2. Antenna Design

**Figure 1:**
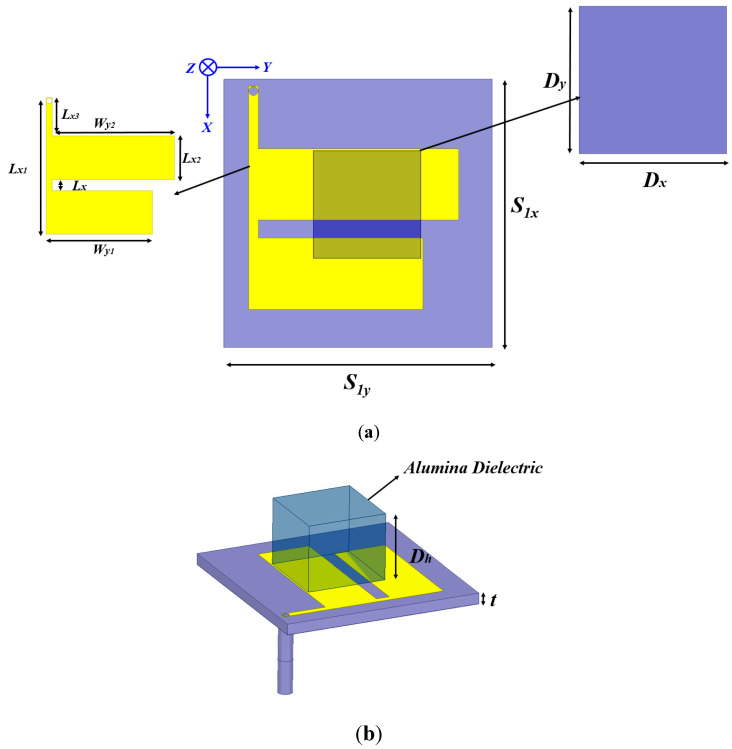
Antenna views, (**a**) top, and (**b**) perspective.

Figure 1 exhibits a designed MIMO antenna. The patch resonator is excited through the transmission line using the co-axial cable. The dielectric resonator (DR) was kept on the patch resonator. The DR is fed by an aperture coupling mechanism. One of the important features of DR is a lack of metal which has huge conducting losses for the GHz frequency spectrum. Customary conductor-based resonators notably suffer from these metallic losses. The DR-based antennas offer extremely high efficiency and hence the high antenna gain. If an appropriate DR material is selected, then DRA provides large bandwidth and gain. Full-wave high-frequency simulation software (HFSS) was employed for the simulation computation. The presented antenna employs alumina material as the dielectric resonator, with ε_r_ = 9.9 and tanδ = 0.0001. This material is widely available and it provides an economical solution compared to other expensive dielectric materials. The aperture-fed DRA was initially kept on the patch resonator. Several repetitions in simulations were taken up to improve the electrical dimensions of the dielectric material to excite the two modes. The fundamental $TE_{01\delta }$ and $TE_{10\delta }$ mode excitations present the key feature to achieving targeted frequency characteristics. Table 1 presents the mechanical sizes of the dielectric resonator.

The electromagnetic fields emanating from the conducting patch are coupled to a dielectric resonator which causes the excitation of the DR. The offset feeding technique is employed for impedance matching. The microstrip feedline dimensions were initially numerically computed and then further optimized for the feeding of the patch antenna. The antenna has been mounted on a standard low-cost FR-4 substrate having a dielectric constant and loss tangent of 4.4 and 0.02, respectively. The 35 μm copper sheets were kept for the design of the patches. The square DR has lateral dimensions of 12 mm and a height of 10 mm. There are few DR-based MIMO antennas in the literature that have more height to attain improved antenna parameters, however, the space constraint may restrict the maximum height dimensions of DRAs. One of the essential reasons to induct a square dielectric resonator instead of a circular/cylindrical dielectric resonator is the mechanical viability in antenna design engineering. The placement optimization of a square DR on the top of the patch is relatively simpler than on cylindrical DRs. It was also observed that it is easier to predict the antenna behavior during the optimization for the case of a square antenna. It is extremely difficult to achieve an antenna numerical model or prediction model for a combination of the patch and dielectric material. It is extremely viable to attain the target antenna parameters through software simulations, however, DRA excitation modes equations can aid in the crude estimation of the antenna resonance. Another reason to avoid circular/cylindrical dielectric resonators is to evade degenerated modes being induced.

**Figure 2:**
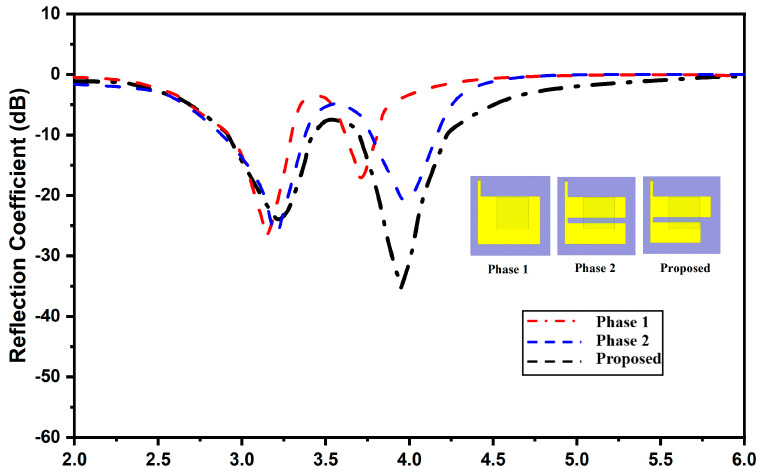
Antenna development phases.

Figure 2 exhibits the antenna evolution. For ‘Phase-1’, the simulated reflection coefficients confirm the impedance bandwidth from 3.3 to GHz and 3.6 to GHz, not covering the desired frequency range. To enhance the bandwidth further and attain the desired resonance, an inverted F-shaped radiator was made by splitting the rectangle into two halves, which is depicted as ‘Phase-2’. This increased the electrical length and thus shifts the cut-off frequency to the upper spectrum and improves the impedance bandwidth. Furthermore, to achieve the desired Sub-6 5G and WiMAX band, an inverted F-shaped antenna is further modified by etching the edge of the first rectangle, as depicted in ‘Phase-3’. The antenna design steps are as follows: (i) implementation of engineered patch resonator, (ii) secondary mode excitation by inducting the dielectric resonator, (iii) altering the antenna resonance by modifying the electrical dimensions of the resonator through slit gap, and (iv) design optimization through sensitivity analysis.

**Figure 3:**
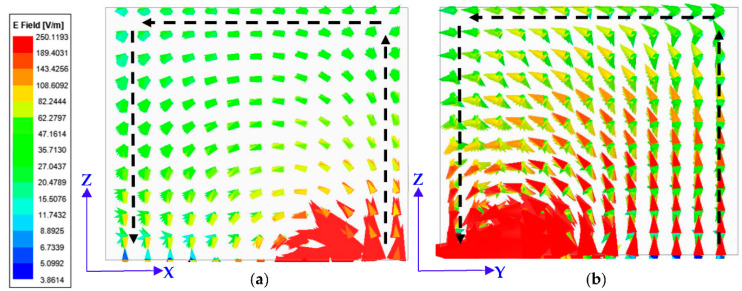

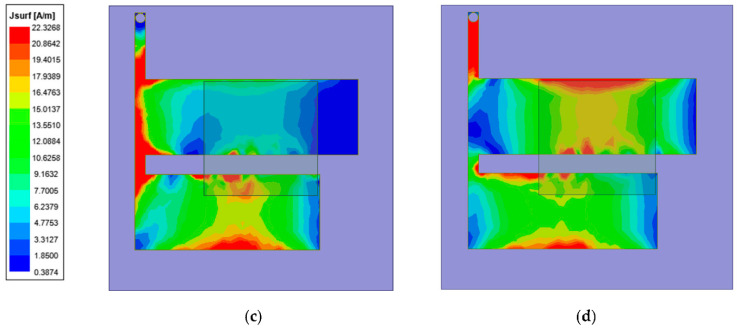
E-Field within DR; (**a**) $TE_{01\delta }$ at 3.3 GHz; (**b**)$\;TE_{10\delta }$ at 3.96 GHz; (**c**) surface current on resonator at 3.3 GHz; and (**d**) surface current at 3.96 GHz.

The calculated sizes of the dielectric resonators were estimated through computation using dielectric resonator equations for the generation of $TE_{01\delta }$ and $TE_{10\delta }\;$modes to achieve the dual-band performance of the resonator. The resonances at 3.3 GHz and 3.9 GHz were attained. Figure 3 illustrates the electric field generated in the DR through the simulation. There is a dissimilarity in the simulated and numerically computed resonance mode as the dielectric wave model does not keep account of the employed feeding mechanism [8].

Through the software simulations, it was possible to visualize the higher-order modes being produced within the DR. The E-field distribution in the x-plane and y-plane of DR at 3.3 GHz and 3.96 GHz are illustrated in Figure 3a,b. Through these figures, the excitation of the $TE_{01\delta }$ and $TE_{10\delta }\;$modes are confirmed. Figure 3c,d depict the surface current density on the resonator.

The resonance and DR mode computations can be done by using the dielectric wave model [8]:

(1) $$k_{z}tan\left(\frac{k_{z}d}{2}\right)=\sqrt{\left(\varepsilon_{r}-1\right)\left(k_{o}{}^{2}\right)-k_{z}{}^{2}}$$

(2) $$k_{x}{}^{2}+k_{y}{}^{2}+k_{z}{}^{2}=\varepsilon_{r}k_{o}{}^{2}$$

(3) $$k_{x}=\frac{m\pi }{a}\;;\;k_{y}=\frac{n\pi }{b};\;k_{o}=\frac{2\pi }{\lambda_{o}}$$

$k_{o\;}$: free-space wavenumber

$k_{x}$,$\;k_{y},$ and $k_{z}$: half-wave variations

*a*, *b*, and *d*: mechanical dimensions.

## 3. Design Optimization

Numerous design parameters of the resonator can alter the antenna resonance and associated radiation performance. The prime parameters include the electrical dimensions of the resonators. Intuitively, modifying the total electrical length alters the resonant modes being excited. A range of techniques to improve antenna radiation parameters by altering the antenna dimensions have been presented in the literature.

**Figure 4:**
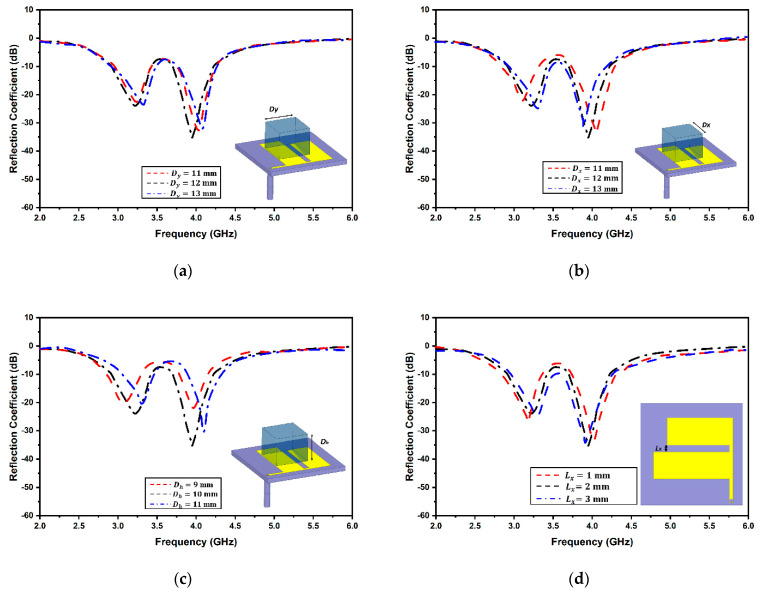

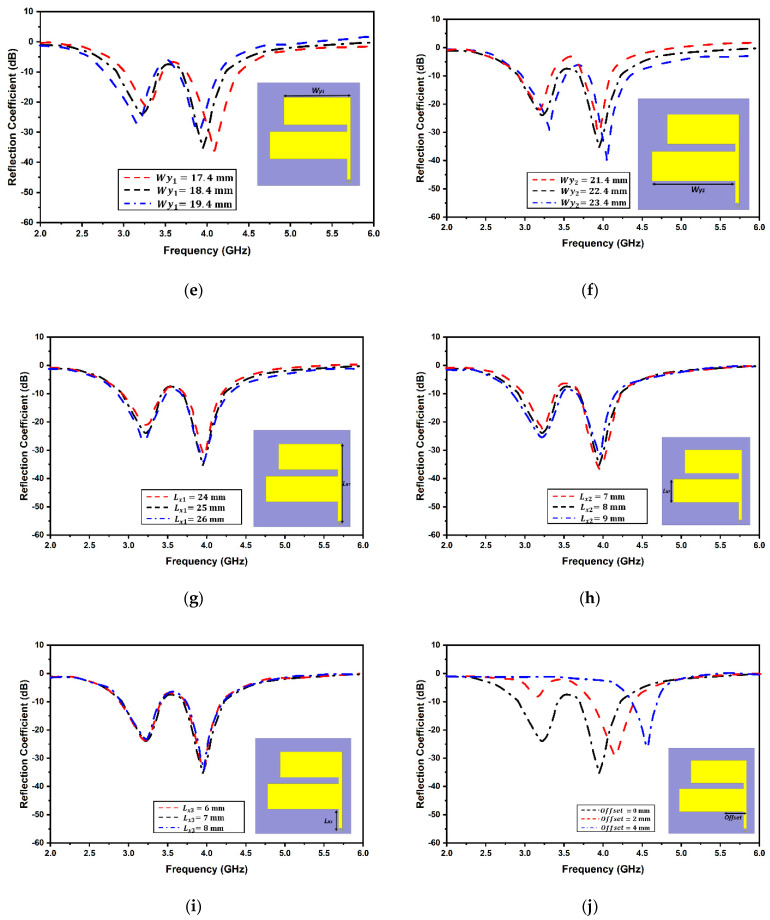
Sensitivity analysis of antenna; (**a**) DR Length D_y_; (**b**) DR Width D_x_; (**c**) DR Height D_h_; (**d**) flare gap; (**e**) upper flare W_y1_; (**f**) lower flare W_y2_; (**g**) length L_x1_; (**h**) width L_x2_; (**i**) length L_x3_; (**j**) offset feed location.

Numerous simulation iterations were performed to achieve the target antenna parameters. Each physical dimension of the antenna was varied to attain improved antenna radiation parameters. Selected parameter variations are displayed in Figure 4. The dielectric resonator dimensions are vital in attaining the desired resonances. The variations in length and width of the resonators are illustrated in Figure 4a,b. Vital changes in resonances occurred while changing the length and width of the DR. It was noticed that by varying the length and width while keeping the other parameters constant, the net electrical current path of the proposed antenna was greatly affected; thereby, the resonant frequencies were shifted. The parametric variation in resonator height is depicted in Figure 4c. It was perceived that reducing the height of the resonator caused the resonance to increase for either band. An additional variation in the gap between the two flares of the antenna is exhibited in Figure 4d. This variation was significant in the change in resonance frequency apart from the variation in dielectric resonator dimensions. It is quite clear from the plot that the resonating frequencies significantly change by altering the flare distance. The impedance matching radically improves with the value of 2mm. The field coupled from the antenna to the dielectric resonator notably varies with a change in gap size which causes alterations in the antenna resonance. Figure 4e,f provide the resonance shift with a change in the length of the upper and lower flares, respectively. It is evident that due to the change in the effective electrical length of the antenna, there is a major shift in the antenna resonance. While the reflection coefficient and bandwidth increase for one of the bands, the other band does not fall on the target frequencies depending on which optimum dimensions are kept to balance the trade-off. A minor variation in reflection coefficient and resonance frequencies was observed for the change in the flare width, as apparent from Figure 4g,h,e. The length variation in Lx3 does not have any effect on the antenna resonance since the impedance does not vary with a minor change in the line length. As evident from Figure 4j, significant modifications were observed with the change in the location of the offset feed due to the modification to the impedance matching of the antenna at the target resonance.

## 4. MIMO Antenna Design Configuration

**Figure 5:**
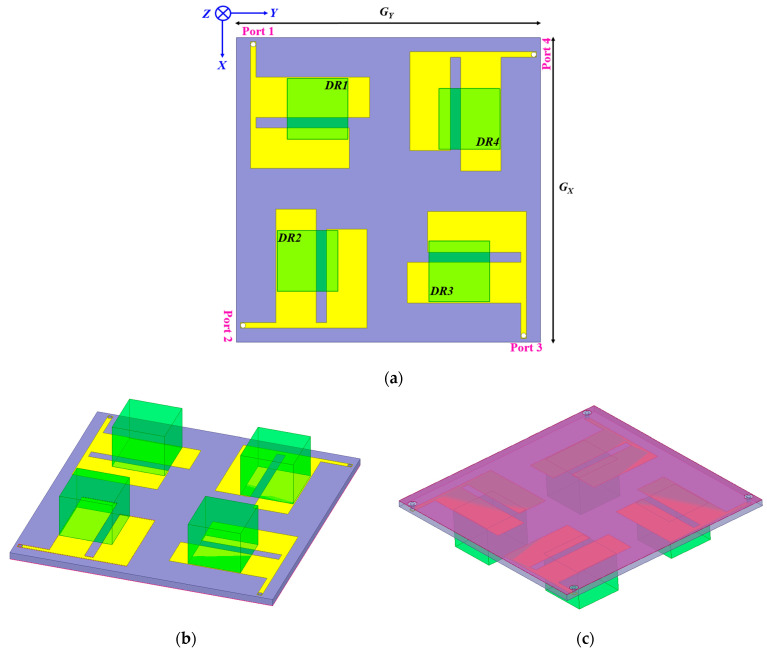
MIMO antenna view; (**a**) top; (**b**) perspective-top; and (**c**) perspective ground.

The proposed MIMO antenna is displayed in Figure 5. The designed MIMO antenna consists of the quad resonating elements of both the dielectric and patch resonators. There needs to be a low correlation between the antenna ports and the low mutual coupling among the four elements for achieving an appropriate diversity performance. Hence, the substrate length and width were modified to the required proportions, keeping the overall electrical dimensions in perspective. The top of the substrate has four identical modified inverted F-shaped radiators placed orthogonally about each other, over which four different alumina dielectric resonators (DR1, DR2, DR3, and DR4) were integrated, whereas, at the back, the full ground profile of the resonator was maintained. Numerous iterative simulations were carried out to achieve electrical compactness without a major trade-off in port isolation. The ground plane and substrate length and width were retained at 60 mm. The overall resonator dimensions are 60 × 60 × 11.6 mm^3^. The dielectric resonators are connected to the patch resonator using conducting adhesive.

## 5. Proposed Antenna Results and Discussions

**Figure 6:**
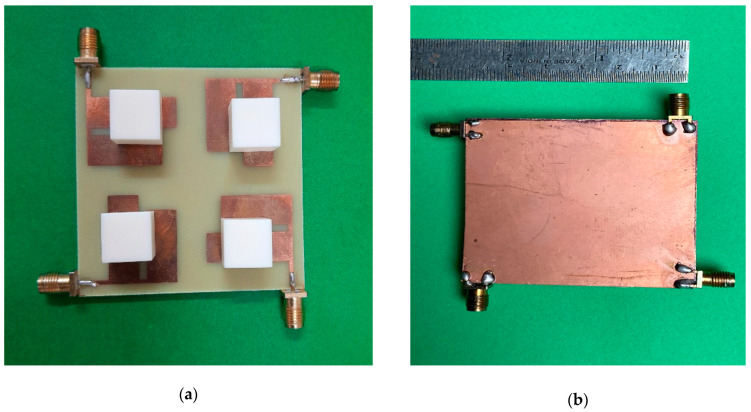
Fabricated DRA Prototype. (**a**) Top View; (**b**) Back View.

**Figure 7:**
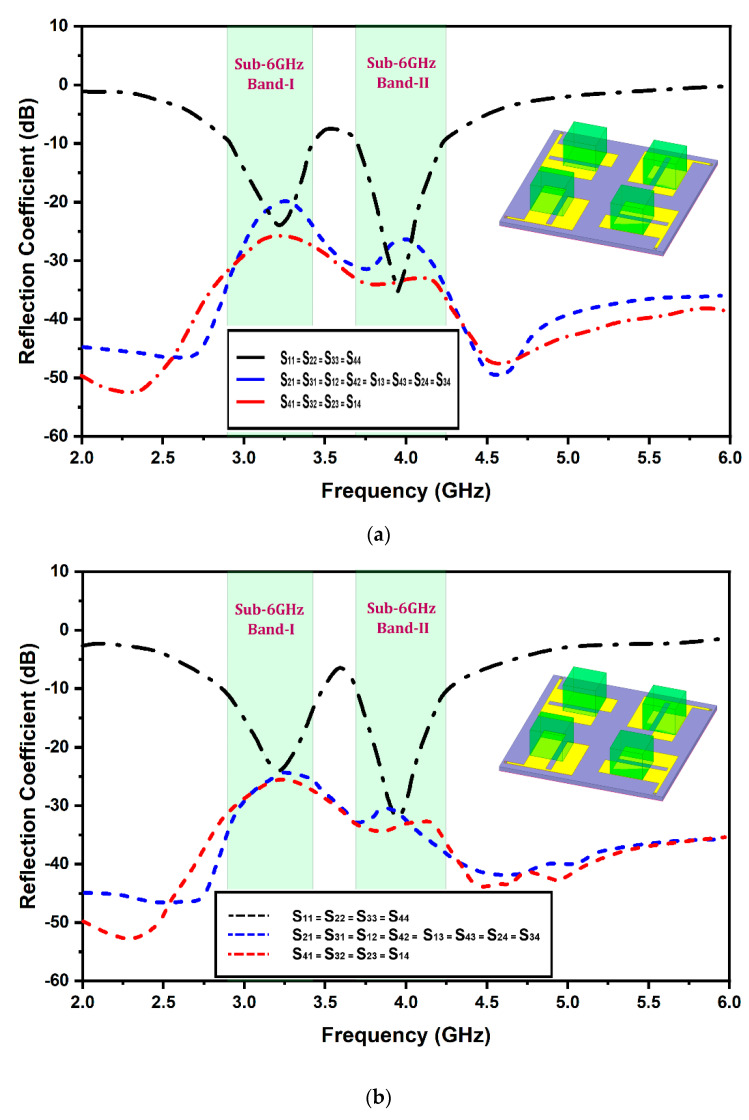
Antenna reflection coefficient; (**a**) simulated; and (**b**) measured.

Figure 6 exhibits the fabricated prototype. The simulated and measured reflection coefficients and mutual coupling plots of the designed Sub-6 5G and WiMAX quad-element MIMO antenna are demonstrated in Figure 7a,b, respectively. A 2:1 VSWR bandwidths of 620 MHz (2.86 GHz–3.48 GHz) and 580 MHz (3.67 GHz–4.25 GHz) are available over two bands of frequencies. However, mutual coupling among ports has been greatly reduced in the presence of DRA compared to without DRA. The proposed structure covers a Sub-6 GHz 5G and WiMAX frequency spectrum with good inter-element isolation.

**Figure 8:**
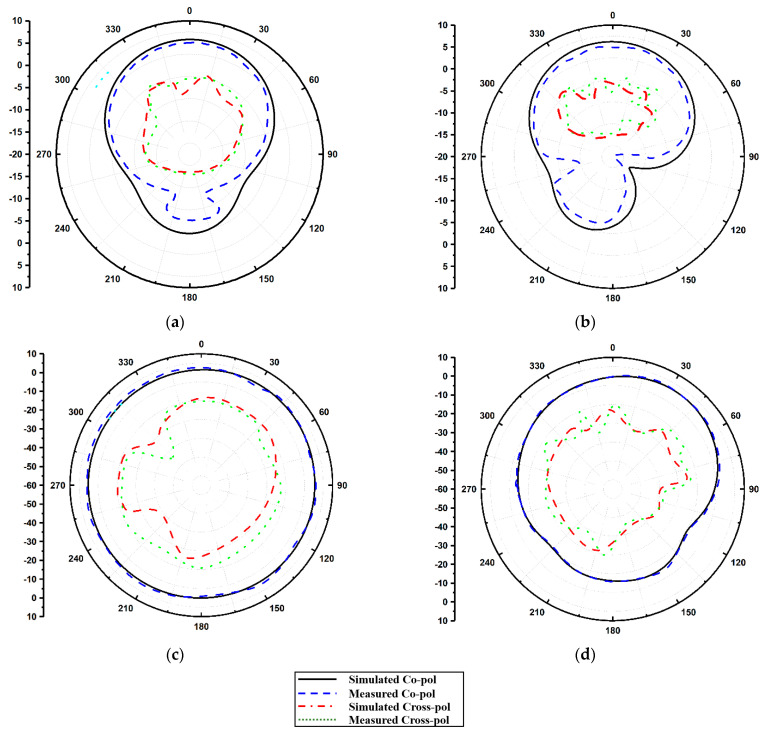
Antenna radiation pattern: (**a**) E-Plane 3.3 GHz, (**b**) E-Plane 3.9 GHz, (**c**) H-Plane 3.3 GHz, and (**d**) 3.9 GHz.

A Keysight E5063A vector network analyzer (VNA) was used to measure the S-parameters of the proposed antenna. These measured results were not the same as the simulated results. The difference in the results was mainly due to manufacturing errors or testing intolerances. From Figure 7, it is also observed that the measured inter-element isolation, i.e., adjacent and diagonal elements, are nearly identical to the simulated isolation results because of the symmetrical structures. The measured isolation is more than −20 dB at 3.3 GHz and less than −26 dB at 3.9 GHz. Furthermore, to validate the proposed antenna’s radiation performance, the simulated 2D radiation patterns were compared with the measured 2D antenna patterns using an anechoic chamber. Figure 8 illustrates the 2D far-field radiation patterns of the proposed antenna at the resonating frequencies of 3.3 GHz and 3.9 GHz, respectively. The 2D radiation patterns were calculated in two planes, both E and H. For measuring the far-field radiation patterns, a single radiating element was fed, whereas all others were matched and terminated with an impedance load of 50 Ω. It is noticed from the plot that because of the symmetry of the design, the patterns are mirror images and are orthogonal to each other. Therefore, the proposed design has great potential in offering a good diversity of characteristics.

**Figure 9:**
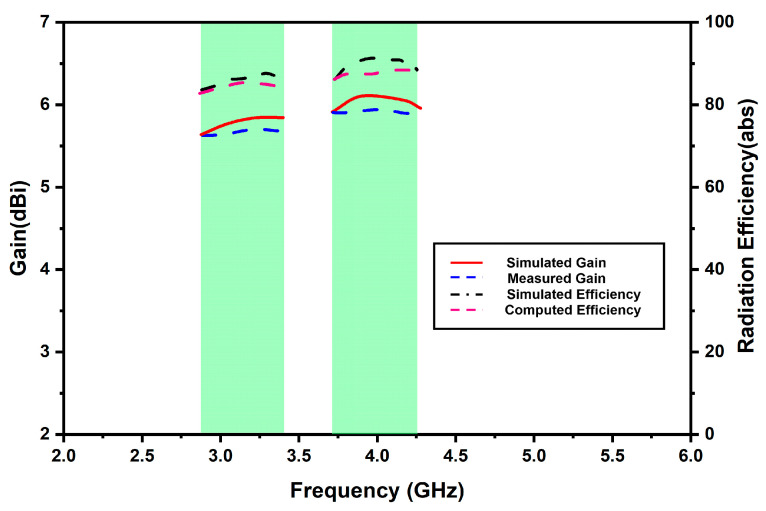
Antenna gain and efficiency against frequency.

Furthermore, Figure 9 highlights the antenna gain. The simulated gains over the operating frequency bands are equal. However, the measured peak gains over the Sub-6 and WiMAX bands are 5.8 dBi and 6.2 dBi, respectively, slightly different from the simulated antenna gains due to fabrication errors. The antenna efficiency for all four radiators is also exhibited in Figure 9. The plot reveals that the peak efficiencies of the proposed antenna over the Sub-6 and WiMAX bands are 88.6% and 90.2%, respectively.

**Figure 10:**
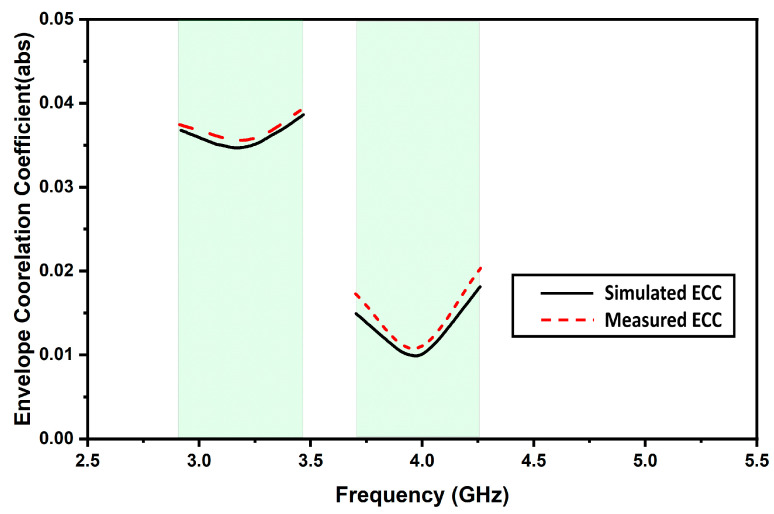
Simulated and measured ECC.

The diversity of the proposed antenna systems for Sub-6 5G and WiMAX applications was computed. Various parameters such as channel capacity loss (CCL), mean effective gain (MEG), and envelop correlation coefficients (ECC) of the radiating elements were analyzed. Firstly, ECC was observed, in which the degree of correlation among antenna elements was measured. ECC should not be more than 0.5. The formula for ECC calculation with far-field is expressed in the following equations [8]:

(4) $$\rho_{e}\left(i,j\right)=\frac{\int_{0}^{2\pi }\int_{0}^{\pi }\;A_{i,j}\left(\theta ,\phi \right)\sin\theta \;d\theta d\phi }{\sqrt{\int_{0}^{2\pi }\int_{0}^{\pi }\;A_{i,i}\left(\theta ,\phi \right)\sin\theta \;d\theta d\phi }+\sqrt{\int_{0}^{2\pi }\int_{0}^{\pi }\;A_{j,j}\left(\theta ,\phi \right)\sin\theta \;d\theta d\phi }}$$

(5) $$A_{i,j}\left(\theta ,\phi \right)=XPRE_{\theta i}\left(\theta ,\phi \right)E^{*}{}_{\theta j}\left(\theta ,\phi \right)P_{\theta }\left(\theta ,\phi \right)+E_{\phi i}\left(\theta ,\phi \right)E^{*}{}_{\phi j}\left(\theta ,\phi \right)P_{\phi }\left(\theta ,\phi \right)$$

(6) $$\rho_{eij}=\frac{{\left|S_{ii}^{*}*S_{ij}+S_{ji}^{*}*S_{jj}\right|}^{2}}{\left(1-{\left|S_{jj}\right|}^{2}-{\left|S_{ji}\right|}^{2})(1-{\left|S_{jj}\right|}^{2}-{\left|S_{ij}\right|}^{2}\right)}$$

where functions *Ai*(*θ*, *ϕ*) and *Aj*(*θ*, *ϕ*) represent the 3D far-field pattern value obtained when port i and j are excited, respectively. Hermitian product operator is denoted using *, and ω refers to the solid angle. XPR is the cross-polarization level defined as the ratio of average power along the phi and theta directions. Figure 10 depicts the simulated and measured ECC plot (for all ports) of the proposed antenna. Both measured and simulated ECC values over Sub-6 5G and WiMAX bands are less than 0.04 and 0.02, respectively. Such low values of ECC for the proposed antenna ensure a high-diversity gain.

**Figure 11:**
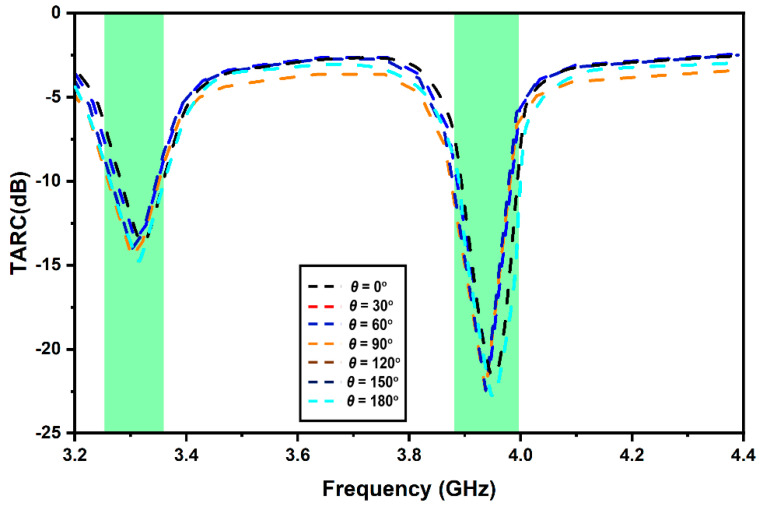
Proposed MIMO antenna TARC.

Moreover, to properly characterize the MIMO system, the total active reflection coefficient (TARC) and channel capacity loss (CCL) were analyzed. TARC provides the reflected–incident power ratio, whereas CCL quantifies the upper threshold amount of data rate where the information signal is continuously transmitting without sustaining any notable error. The optimal value of TARC is –22.46; the TARC is shown in Figure 11. The TARC can be calculated as [8]:

(7) $$TARC=\frac{\sqrt{\sum_{n=1}^{N}{\left|b_{n}\right|}^{2}}}{\sqrt{\sum_{n=1}^{N}{\left|a_{n}\right|}^{2}}}$$

(8) $$b_{n}=\left[S\right]a_{n}$$

where *b_i_* represents the reflected signal, *a_i_* represents the incident signal, *N* depicts the number of elements in the MIMO system, and *S* denotes the scattering parameter.

**Figure 12:**
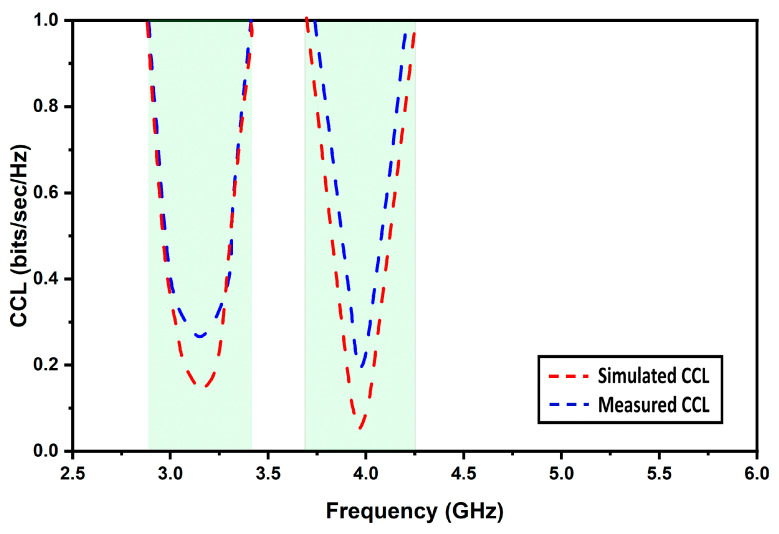
Simulated and measured CCL.

Thus, ideally, channel capacity loss (CCL) must be zero. Figure 12 depicts CCL. The following expression is used to compute CCL [8]:

(9) $$C_{loss}=-log_{2}\left|\psi^{R}\right|\;\;\;$$

The Ψ^R^ represents the correlation matrix between the antenna elements at the receiver. It is described as follows in Equation [8]:

(10) $$\Psi^{R}=\left[\begin{array}{cccc} \rho_{11} & \rho_{12} & \rho_{13} & \rho_{14}\\ \rho_{21} & \rho_{22} & \rho_{23} & \rho_{24}\\ \rho_{31} & \rho_{32} & \rho_{33} & \rho_{34}\\ \rho_{41} & \rho_{42} & \rho_{43} & \rho_{44} \end{array}\right]$$

(11) $$\rho_{ii}=1-\sum_{n=1}^{4}\left(S_{in}{}^{*}S_{ni}\right);\rho_{ij}=-\sum_{n=1}^{4}\left(S_{in}{}^{*}S_{nj}\right)\;\mathrm{For}i,j=\mathrm{1,2,3,or 4.}$$

The desired level of CCL should not be more than 0.4 bits/sec/Hz. It is visible from the plot that the CCL values are lower than the expected values over both the Sub-6 5G and WiMAX bands of frequencies, which confirms that the proposed MIMO antenna offers stable diversity characteristics.

Another important diversity parameter is MEG, which measures the average received signal strength of each element. The mathematical expression for MEG calculation is given as [8]:

(12) $$MEG=\int_{0}^{2\pi }\int_{0}^{\pi }\;\left(\frac{XPR}{1+XPR}G_{\theta }\left(\theta ,\phi \right)P_{\theta }\left(\theta ,\phi \right)+\frac{1}{1+XPR}G_{\phi }\left(\theta ,\phi \right)P_{\phi }\left(\theta ,\phi \right)\right)\sin\theta \;d\theta d\phi$$

(13) $$MEG_{i}=0.5\left[1-\overset{N}{\underset{j=1}{\sum }}{\left|S_{ij}\right|}^{2}\right]$$

**Figure 13:**
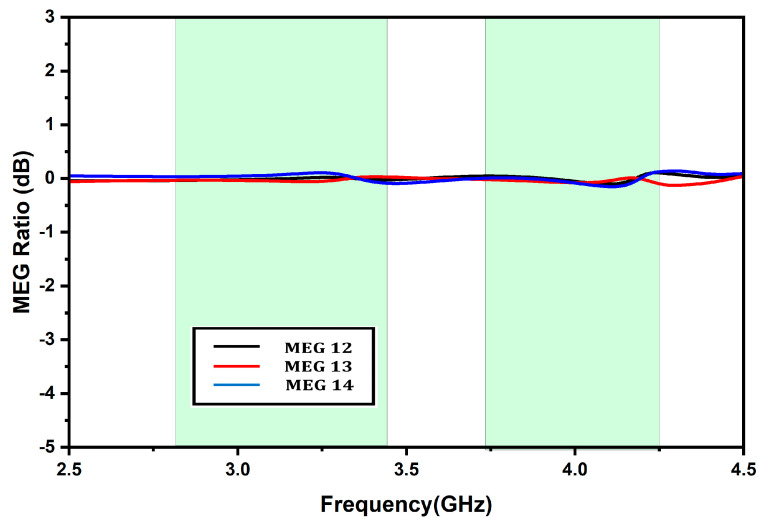
Mean effective gain.

XPR is the cross-polarization level defined as the ratio of average power along the phi and theta directions, where i ranges from 1 to 4, since the proposed antenna has four ports, and $\left|S_{ij}\right|$ depicts the scattering parameters of the proposed SIW-fed DRA MIMO antenna. Figure 13 presents the MEGs for all ports of the proposed antenna over Sub-6 5G and WiMAX bands. Here MEG12 (between Port 1 and Port 2) and MEG14 (between Port 1 and Port 4) represent the MEG of the adjacent elements, whereas MEG13 (between Port 1 and Port 3) represents the diagonal elements. It is visible from the plot that the MEGs ratio is almost equal and close to unity. Hence, the proposed antenna fulfills the equality condition of the diverse systems and consequently exhibits excellent MEG characteristics, since the deviations among the MEGs are well under 3dB. Table 2 exhibits the antenna comparison with the selected literature, and evidently, the presented antenna demonstrates better characteristics.

## 6. Conclusions

A dielectric resonator-based four-port MIMO antenna is presented. Optimized electromagnetic coupling of fields emanating from patch resonators and fed to the dielectric material induces the dual-mode operation of the antenna. The $TE_{01\delta }$ and $TE_{10\delta }$ modes were excited to achieve dual resonances for target applications. The antenna provides a high gain of 5.8 and 6.2 dBi at 3.3 GHz and 3.9 GHz, respectively. The presented diversity performance of the antenna meets the desired communication applications.

## Figures and Tables

**Table 1:** Antenna Mechanical Dimensions.

Parameter	Dimensions (mm)	Parameter	Dimensions (mm)
*S_1x_ = S_1y_*	30	*Lx3*	7
*Dx = Dy*	12	*Wy1*	19.5
*Lx*	2	*Wy2*	22.4
*Lx1*	25	*Dh*	10
*Lx2*	8	*t*	1.6

**Table 2:** Comparison of the proposed antenna with the literature.

Reference	Operating Frequencies (GHz)	Antenna Size (λ)	Isolation (dB)	Gain (dBi)	Fractional Bandwidth (%)	Efficiency (%)	Feed Type
[33]	4.9	2.28 × 0.73 × 0.13	25	6.2	5	--	Microstrip
[34]	3.22–3.97, 4.95–5.51	0.86 × 0.86 × 0.12	18, 20	5.2, 5.5	4.9, 2.3	94	Microstrip
[35]	5.71–8.2, 7.57–7.95	1.53 × 1.53 × 0.12	20, 15	−1.9, 3.8	34.8, 4.5	--	Microstrip
[36]	3.40–4.13	1.13 × 1.13 × 0.19	14	8.1	19.4	91	Probe
[37]	3.50–5.10	1.43 × 1.43 × 0.36	--	8.5	46	89	Probe
[38]	4.56–9.96	1.5 × 1.5 × 0.39	23	--	73.9	--	Trapezoidal Patch
[39]	4.33–7.02	2.6 × 2.6 × 0.36	--	--	48	90	Probe
Proposed Antenna	2.86–3.48 3.67–4.25	0.66 × 0.66 × 0.12	20, 26	5.8, 6.2	18.7, 14.6	88.6, 90.2	Aperture Couple

## Data Availability

Not applicable.
